# Perceived Stress, Salivary Cortisol, and Temperament Traits among Students of Dental Medicine: A Prospective and Interventional Study

**DOI:** 10.3390/bs14040289

**Published:** 2024-03-31

**Authors:** Bruno Špiljak, Luka Šimunović, Maja Vilibić, Milena Hanžek, Danijel Crnković, Liborija Lugović-Mihić

**Affiliations:** 1 School of Dental Medicine, 10000 Zagreb, Croatia; bruno.spiljak@gmail.com; 2 Department of Orthodontics, School of Dental Medicine, 10000 Zagreb, Croatia; lsimunovic@sfzg.hr; 3 Department of Psychiatry, University Hospital Center Sestre Milosrdnice, Vinogradska cesta 29, 10000 Zagreb, Croatia; maja.vilibic@gmail.com; 4 School of Medicine, Catholic University of Croatia, Ilica 242 ulaz iz Domobranske ulice, 10000 Zagreb, Croatia; 5 Department of Clinical Chemistry, University Hospital Center Sestre Milosrdnice, 10000 Zagreb, Croatia; milenanjegovan13@gmail.com; 6 Department of Psychiatry, University Hospital Center Sestre Milosrdnice, Academy of Music, 10000 Zagreb, Croatia; crnkovic.danijel@gmail.com; 7 School of Dental Medicine, University Hospital Center Sestre Milosrdnice Vinogradska cesta 29, 10000 Zagreb, Croatia; 8 Department of Dermatovenereology, University Hospital Center Vinogradska cesta 29, 10000 Zagreb, Croatia

**Keywords:** academic stress, cortisol, PSS, temperament, PMR

## Abstract

Academic stress affects students’ psychological and physiological well-being. Dental undergraduate programs are known for their demanding curriculum, leading to significant stress symptoms. The objective was to determine if salivary cortisol levels were higher in students exposed to academic stress, assess the relationship between stress severity/temperament and cortisol values, and explore relaxation technique effects. Salivary cortisol was measured at two time points for all participants: Before exams and during a relaxation period after summer break. A third measurement was conducted for students with high pre-test cortisol levels who received instructions on progressive muscle relaxation (PMR) before subsequent exams. Additionally, participants completed two questionnaires: Perceived Stress Scale (PSS) and Fisher’s Temperament Questionnaire. The group analysis based on the PSS indicated that 39 participants reported high stress. Women demonstrated significantly higher stress than men (*p* = 0.042054). A significant difference in stress levels was observed between director and builder temperament types (*p* = 0.029276). Cortisol levels showed a significant decrease from the first measurement to the second measurement, and the third measurement after implementing PMR. The grade in the “Dermatovenereology” course correlated with stress level according to the PSS (k = 0.578467). Pre-test cortisol levels correlated with the frequency of using PMR guidelines during winter test periods (k = 0.416138). Stress negatively affects the immune system and poses health risks. Implementing stress reduction techniques in dental/medical education could benefit students and the healthcare system.

## 1. Introduction

Academic stress (AS) is a significant, everyday stress that impacts students’ psychological and physiological health. Exams, in particular, are considered one of the most pronounced sources of stress among students [1,2,3]. Although the results of studies differ, a significant association between AS and aspects of the immune system has been observed, including a decreased immune response. The heterogeneous results of the various studies probably reflect the insufficient study of the impact of AS itself on students, without looking at other associated factors (high professional competitiveness, lack of time for relaxation and communication with friends and family, etc.) [2,3,4]. Medical and dental faculties are stressful environments for most students; undergraduate programs are often associated with significant symptoms of stress because they are among the longest and most demanding programs [5,6,7]. The most significant causes of AS in these students are exams and clinical exercises [5]. Medical and dental students more often notice, manifest, and report increased anxiety, depression, obsessive-compulsive behaviors (absent the disorder), and increased interpersonal sensitivity, among other symptoms [5,6,7]. After enduring long-term academic stress, doctors and dentists may then experience professional stress caused by various factors, such as interactions with patients and other staff, fear of physical violence when working with certain patients, malpractice lawsuits, and other financial concerns [7]. Cortisol plays a key role in the body’s response to stress [8]. In routine diagnostics, cortisol is most commonly measured in the plasma, which requires a blood sample, an invasive method potentially causing additional stress and discomfort [9]. Cortisol can also be measured in the saliva by collecting samples in a sterile container, or by using absorbent materials (so-called Salivette systems), without causing undue stress. This easy and non-invasive method of collecting samples is being increasingly used to measure cortisol [10,11]. Numerous studies have confirmed that the analysis of salivary cortisol, as a stress biomarker, is a reliable alternative to analyzing cortisol in the blood and urine [12,13]. Salivary cortisol is a biologically active form of free cortisol. It has a concentration ten times lower than total serum cortisol and correlates well with plasma cortisol (adult correlation is about 0.75)—this correlation remains high throughout the circadian cycle [14]. Cortisol secretion is affected by the circadian rhythm, with the lowest fluctuations expected in the late afternoon and the highest concentrations of cortisol expected in the morning between 7 and 10 a.m. (The specific time a person wakes has no major influence on the morning cortisol response, and within 30 min after waking, the free cortisol level rises from 50% to 75%) [15]. Salivary cortisol values are affected by a number of factors, including during the body’s response to stress: Age and gender, endogenous and exogenous sex hormone values (e.g., menstrual cycle, oral contraceptive use, hormone replacement therapy, pregnancy, breastfeeding), certain medications (psychopharmaceuticals, corticosteroids), smoking, energy-rich diets, coffee and alcohol consumption, and exercise [16]. In addition, it is assumed that salivary cortisol, during stress, is influenced (at least partially) by stable factors such as character traits, although the results from previous studies are not uniform [13,17,18,19]. Numerous studies have suggested that there are benefits to using cortisol as a salivary biomarker and as an indicator of AS [20,21]. Significantly higher cortisol values were measured in students just before and during exam periods, while significantly reduced values were found after exam periods [22,23,24]. However, the existing literature also shows some conflicting results in the relationship between academic stress and salivary cortisol levels [25]. Recent literature has explored the intricate relationships between temperament traits, anxiety, and cortisol levels among students, highlighting the complexity and multidimensional nature of these associations. One study demonstrated significant associations between temperament dimensions such as effortful control and negative affectivity with attention-deficit/hyperactivity disorder (ADHD), but not directly with awakening cortisol levels, suggesting the nuanced role of temperament in physiological stress responses [26]. Further research has revealed the significant impact of social and environmental factors, such as teachers’ personality traits, on student anxiety levels, indicating the influence of external factors on internal psychological states [27]. Moreover, temperament has been shown to play a critical role in emotional regulation and susceptibility to psychiatric conditions, including symptoms of anxiety and depression, emphasizing its importance in mental health [28]. Additionally, the link between temperament and school readiness has been explored, revealing how temperament traits can affect academic and social readiness, potentially influencing anxiety levels through mechanisms such as academic performance and peer interactions [29]. These studies collectively underscore the multifaceted interplay between temperament traits, anxiety, and physiological stress markers, highlighting the need for further research to better understand these dynamics and to inform targeted interventions for anxiety and stress management among students. Therefore, we wanted to examine salivary cortisol levels in dental medicine students exposed to AS in relation to perceived stress levels (measured by stress questionnaires). We also looked at the possible correlation between salivary cortisol values and perceived stress severity in the context of personal temperament, also determined by questionnaires. Finally, we looked at relaxation methods as a way to reduce students’ stress during exams.

## 2. Materials and Methods

**Figure 1:**
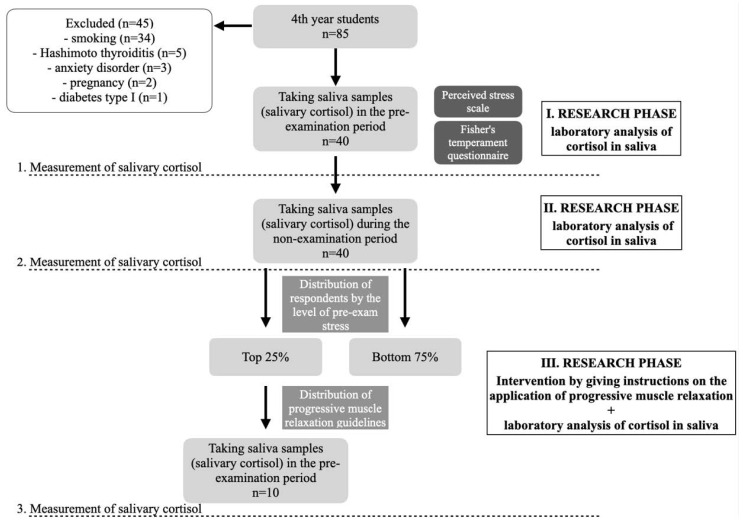
Hodogram (a simplified roadmap) of the prospective and interventional research.

This prospective and interventional study was conducted with students of dental medicine during exams and classes and was conducted at the Sestre Milosrdnice University Hospital Centre (SMUHC), at the Department of Dermatovenereology and the Department of Clinical Chemistry, Zagreb, Croatia. The research was conducted in accordance with the basic principles of the Helsinki Declaration (as revised by the World Medical Association Declaration of Helsinki 2013) and was approved by the Ethics Committees of SMUHC and the School of Dental Medicine, Zagreb, Croatia (class: 003-06/21-03/016 and 05-PA-30-XXIX-9/2021). We followed dentistry students, all in the same year of study, and measured their pretest salivary cortisol values immediately before exams, and compared those values with the values measured after a longer, relaxed non-exam period. We compared these levels by sex and temperament characteristics. We examined indicators of perceived stress using the Perceived Stress Scale (PSS) and compared PSS levels of perceived stress (low, medium, or high) with pretest salivary cortisol levels. In addition, we investigated the correlation between temperament characteristics (determined by the Fisher Temperament Inventory, or FTI) and stress level (determined by PSS values and pre-test salivary cortisol values). Afterward, we applied the guidelines of progressive muscle relaxation (PMR) to students with the most pronounced stress values (measured at the first exposure to pre-test academic stress) to examine whether/how much the use of PMR affected salivary cortisol values during subsequent academic stress. This study included prospective monitoring of cortisol values during individual time points (Figure 1). Salivary cortisol levels were measured at 2 time points for all students: (1) Just before the exam; and (2) after the relaxation period (summer vacation). When cortisol was initially measured (at the first time point), subjects were also given questionnaires about perceived stress (PSS) and temperament (FTI). Additionally, due to laboratory constraints at that time, only those students with high pretest cortisol (at the first pretest measurement, 10 respondents) were measured a third time, wherein after the second measurement they received instructions on how to practice PMR. All participants confirmed using PMR before the last exam (third time point), at which time cortisol values were again measured.

### 2.1. Respondents

This study included 40 subjects (male = 12; female = 28) students of the School of Dental Medicine, University of Zagreb, aged 20–25 years (to prove statistical significance α = 0.05 statistical power 80%, two-tailed t-test for matched pairs, had to include at least 34 subjects). The inclusion criteria for the study were that participants had to be in their 4th year of study, be aged 18 or more, and sign an informed consent prior to any study procedure. Exclusion criteria were recent use of systemic corticosteroids or immunosuppressive agents (one month prior to study enrollment); having a previously diagnosed psychiatric disorder; chronic smoking and alcohol consumption; pregnancy; having a systemic immune, endocrine or malignant disease (Figure 1). 

### 2.2. Methods

#### 2.2.1. Saliva Collection Method and Salivary Cortisol Analysis

The first salivary cortisol measurement was taken before subjects took the oral dermatovenereology test (held at SMUHC), based on multi-year student surveys indicating it is one of the most demanding and difficult exams. For all students, salivary cortisol was determined by taking samples of saliva in the morning (9–11 a.m.) to prevent values from being affected by the daily oscillation of cortisol levels. Before taking the sample, subjects rinsed their mouth thoroughly with water to clean the oral cavity, then waited 10 min. The procedure involved placing absorbent material (cotton pieces) in the mouth, the so-called Salivette system (Sarstedt, Nümbrecht, Germany), on the parotid gland outlet for 5 min. Samples were then centrifuged, and the supernatant was stored in a refrigerator (FRYKA-Kältetechnik GmbH, Esslingen, Germany) at −80 °C until analyzed. Samples were analyzed by the ELISA (enzyme-linked immunosorbent assay) test by EUROIMMUN (Medizinische Labordiagnostika AG, Lübeck, Germany), at the Department of Clinical Chemistry of SMUHC. The use of this method for measuring cortisol in saliva was confirmed by the manufacturer with the following specifications: Limit of detection, 0.15 ng/mL; variation within the test, 3.7%; and variation between tests, 7.4% [30]. To carry out salivary cortisol analysis, murine anti human antibodies were used, and the specific incubation/reaction of antibodies and cortisol was carried out at room temperature (from +18 °C to +25 °C). The reference average cortisol concentrations were measured by the above-mentioned manufacturer’s test, and their values were compared with the ELISA reference average for cortisol [13].

#### 2.2.2. Measuring Stress Using the Perceived Stress Scale (PSS) and Determining Temperament Using the Fisher Temperament Inventory (FTI)

Prior to the exam, participants had also completed the Perceived Stress Scale (PSS) [31] as a measure of their stress levels. After the exam, an email was sent to each participant with their demographic data (name, gender, year of birth), and FTI questionnaire [32], as well as a question about their exam results/grade. The FTI was measured afterward, given that temperament does not change, regardless of whether it is during an exam period or not. Temperament refers to a set of biologically based traits that emerge early in life and demonstrate significant consistency into adulthood [33]. These traits are not altered by stress, hence no significant differences in FTI scores are expected whether the inventory is administered before (pre-test) or after (post-test) a stress-inducing event [33,34]. Originally conceived in 1983, the PSS is a scale that is a common instrument for assessing mental stress. It provides insight into how different situations affect respondents’ feelings and perceptions of stress. It consists of 10 questions answered on the Likert scale (0 = never, 4 = very often) that provide answers about the feelings and thoughts of respondents during the last month [31]. Individual PSS scores can range from 0 to 40, with higher scores reflecting higher perceived stress (scores: 0–13 = low stress, 14–26 = moderate stress, 27–40 = high stress). The FTI [32] examines four dimensions of human temperament related to the functioning of particular hormonal and neurotransmitter systems. The four dimensions of temperament are curious/energetic (*explorer*), careful/according to social standards (*builder*), analytical/decisive (*director*), and prosocial/empathetic (*negotiator*). According to the questionnaire’s author, Helen Elizabeth Fisher, each individual is a unique “mixture” of all four dimensions and it is possible for individuals to express only some dimensions of temperament more often. There are four possible answers for each question (strongly disagree, disagree, agree, and strongly agree). The result for each scale is formed by summing the points achieved on the individual plots of the scale, with the answer “strongly disagree” on all questions being scored with zero points; the answer “disagree” scored with one point; the answer “agree” with two points; and the answer “strongly agree” with three points. Results on each scale range from 0 to 42 points, where higher results indicate a more pronounced dimension of temperament. Results indicate a primary temperament type, the scale achieving the highest number.

#### 2.2.3. Evaluation of Questionnaires, Follow-Up Procedure and Other Cortisol Measurement

The questionnaires were used as assessment tools for stress (PSS) and as an instrument for assessing the primary type of temperament of each subject individually (FTI), therefore describing parameters that could affect their quality of life. Based on PSS results, students were divided into three groups: Low, medium, and high levels of perceived stress. Students were also divided into the four types of temperament (*explorer*, *builder*, *director*, and *negotiator*) as indicated by FTI results. A second saliva sample was then taken from all subjects (to assess cortisol), this time in a relaxed period without exams (at the very beginning of a new semester and class). Additionally, each participant’s exam grade was recorded, where the best grade was excellent, five, on a range of 1–5.

#### 2.2.4. Third Cortisol Measurement following Progressive Muscle Relaxation (PMR)—And Its Use in Subjects with a Third Cortisol Measurement

After the second set of saliva samples was collected (taken after the relaxed period—during the period without exams, 4 months after collecting the first set of saliva samples), each student received a document with instructions on PMR and was asked to carefully study them and try to apply them before the next exam period comprised of the subject with the same European Credit Transfer and Accumulation System (ECTS) points, since subjects with the same ECTS points are considered to have an equivalent level of difficulty and workload [35,36]. PMR is a relaxation technique developed in the 1920s by Edmund Jacobson that helps relieve the negative effects of stress and control anxiety [37]. It consists of a series of stretching and relaxing exercises for different muscle groups for the whole body, thus achieving a relaxed state of body and mind. In a continuous sequence of exercises, the various muscle groups contract and relax [38]. It has been shown to be successful in healthy, stress-exposed individuals and in people with mental and physical health problems [39]. Relaxation regulates the heartbeat, slows breathing, lowers blood pressure, reduces muscle tension, and promotes a psychological experience of whole-body relaxation. Exercises are intended for anyone, both healthy individuals and those with mental health issues—anyone exposed to different types of tension or stress. Due to laboratory constraints at that time, only the study participants (10 respondents) with the highest recorded academic stress (as indicated by cortisol values) were asked to provide a third saliva sample. This sample was taken just before their next exam (of a similar type) 4 months after the students had been instructed in relaxation exercises, in order to assess the impact of PMR application on academic stress. To gain insight into the effect of PMR, all subjects were forwarded a new questionnaire containing 4 questions about PMR (answers given on the Likert scale [1 = never; 5 = very often]) and about how useful its application was personally. Participants evaluated the PMR guidelines, their application during examination deadlines, how much they helped to cope with academic stress, and how they influenced academic achievement (in the form of better grades).

#### 2.2.5. Statistical Analysis

The research results are presented in the form of boxplots with median values and interquartile range. Measurements of parameters (salivary cortisol, PSS) were compared separately for each group by gender and Fisher’s temperament types. Comparisons were evaluated by the Mann–Whitney U test with continuity correction for comparison between genders, Kruskal–Wallis ANOVA by ranks test with post-hoc Dunn’s multiple p comparison test for comparison between Fisher’s temperament types, the Wilcoxon rank test and Friedman’s ANOVA with Kendall’s W (coefficient of concordance) for repeated measurements of salivary cortisol levels, since the changes of measured parameters did not follow the normal distribution, which was confirmed by the normality test—Shapiro-Wilk and Kolmogorov tests for each parameter. Also, indicators of asymmetry and curvature indicated an abnormal distribution. The Spearman rank test was used for the analysis of correlations between parameters. Results were considered statistically significant at 0.05. Analysis was carried out using the Statistica software package (TIBICO Statistica Version 14.0.0.15).

## 3. Results

### 3.1. Analysis of the Perceived Stress Scale (PSS) Results in Relation to Other Parameters/Variables

**Figure 2:**
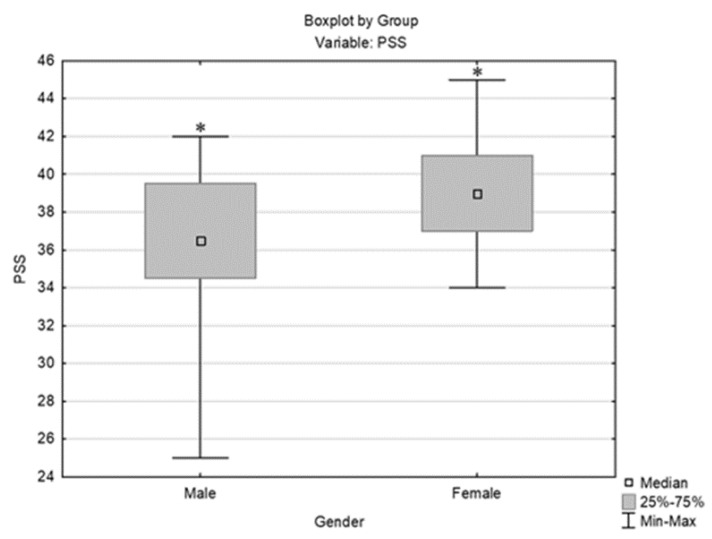
Comparison of stress levels (PSS) between genders; * indicates statistically significant data [Mann–Whitney U test, U = 99.5, Z adjusted = −2.021, 2*1 sided exact *p* = 0.042].

**Figure 3:**
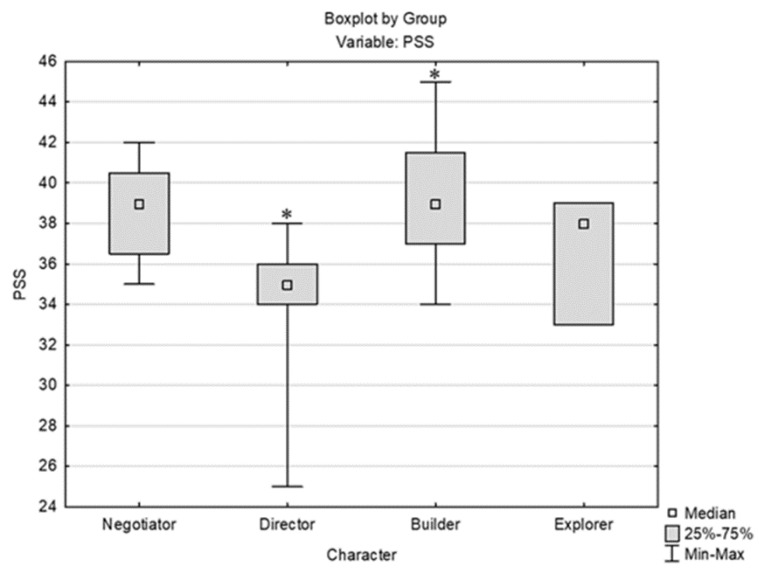
Comparison of stress level (PSS) by temperament (FTI); * indicates statistically significant data [Kruskal–Wallis test: H (3, N = 40) = 8.933, *p* = 0.0302; post hoc Dunn’s test *p* = 0.029].

PSS levels, as measured immediately after the first exam, showed high perceived stress in almost all students (39/40), with medium stress in one student. There were no subjects with low stress. Since the majority of examinees (97.5% of respondents) had high stress, the respondents were not consequently separated by PSS score. According to descriptive statistics and a comparison of stress levels (per PSS) between men and women (Figure 2), significantly higher stress levels were observed in women than in men (Mann–Whitney U test, U = 99.5, Z adjusted = −2.021, 2*1 sided exact *p* = 0.042). According to temperament type (per FTI), there was a significant difference in the stress level (per PSS) between *director* and *builder*, where *builder* had significantly higher stress than *director* (*p* = 0.029) (Figure 3).

### 3.2. Results Obtained by Salivary Cortisol Analysis

**Figure 4:**
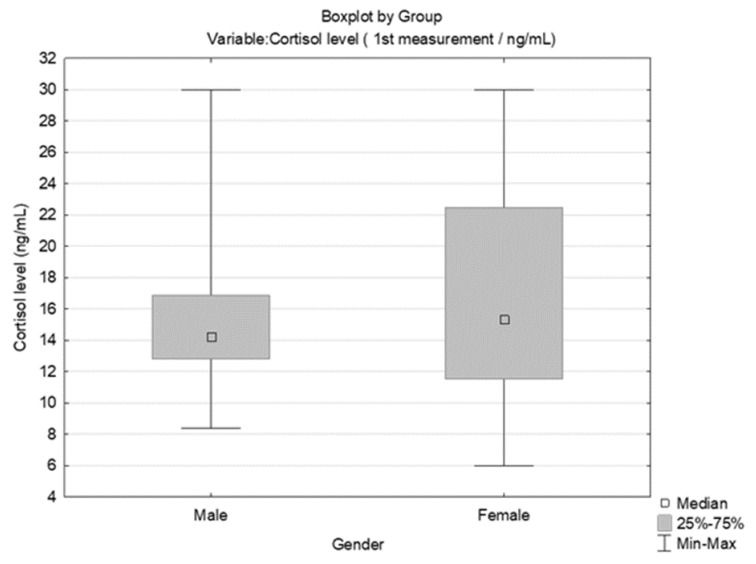
Comparison of pre-test cortisol levels (ng/mL) between genders [Mann–Whitney U test, U = 139.5, Z adjusted = −0.827, 2*1 sided exact *p* = 0.405].

**Figure 5:**
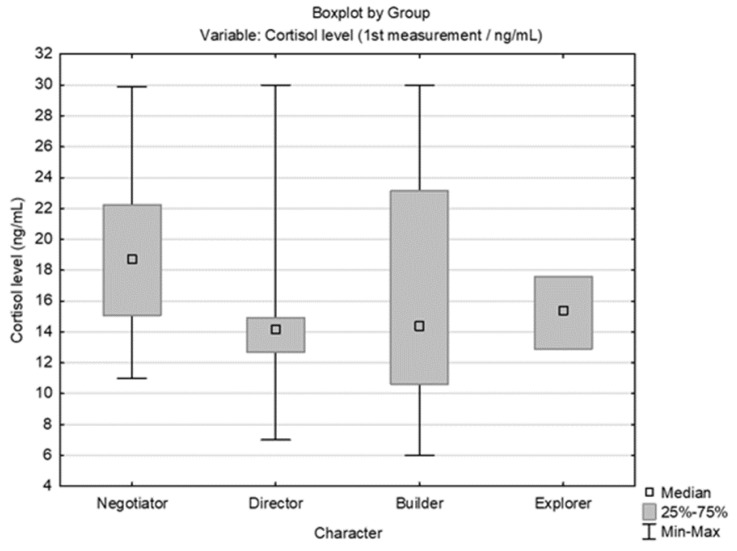
Comparison of pre-exam cortisol levels (ng/mL) by the Fisher scale of temperament types [Kruskal–Wallis test: H (3, N = 40) = 2.335, *p* = 0.5058].

**Figure 6:**
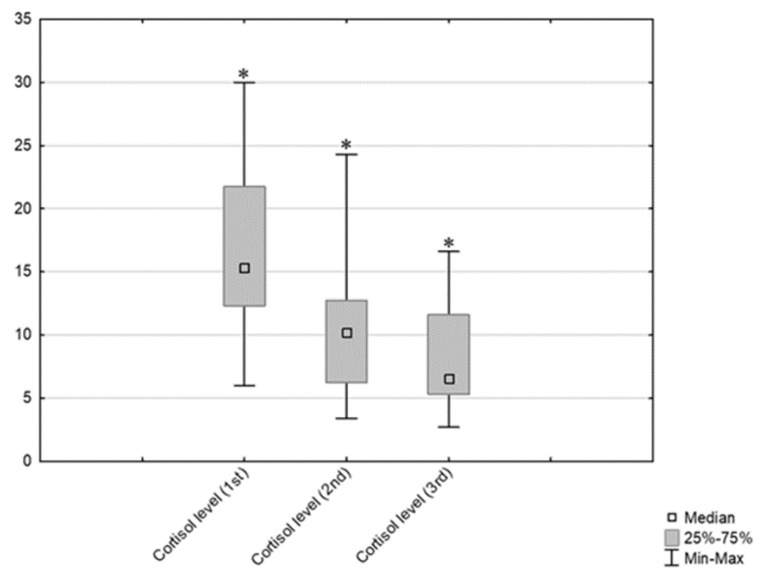
Comparison of the three cortisol measurements (pre–exam; non–exam period after summer vacation; and after PMI (25% of students who had the highest cortisol level at first measurement) * indicates statistically heterogenous data, *p* < 0.05 (Wilcoxon match paired test) [Friedman ANOVA and Kendall coeff. of concordance; ANOVA Chi Sqr. (N = 10, df = 2) = 20.00 *p* = 0.00005; coeff. of concordance = 1, Aver. rank r = 1].

According to descriptive statistics and a comparison of students’ pre-test cortisol levels by sex, there was no significant difference between genders (Figure 4). However, there was a significant difference between the first (pre-exam) and second (non-exam period) cortisol measurements (ng/mL) for both males (Wilcoxon matched pairs test, N = 12, T = 0.00, Z = 3.059, *p* = 0.002) and females (Wilcoxon matched pairs test, N = 28, T = 0.00, Z = 4.623, *p* < 0.001). When comparing cortisol and temperament types (by Fisher’s division), no significant difference in pre-exam cortisol levels was observed. The comparison is presented in Figure 5. A comparison of the values for the three different cortisol measurements (pre-exam; post-exam at the end of summer vacation; and just before the exam after progressive muscle relaxation [25% of students with the highest cortisol level at the first measurement]) is presented with a graphic representation in Figure 6.

### 3.3. Exam Scores and Correlations

**Figure 7:**
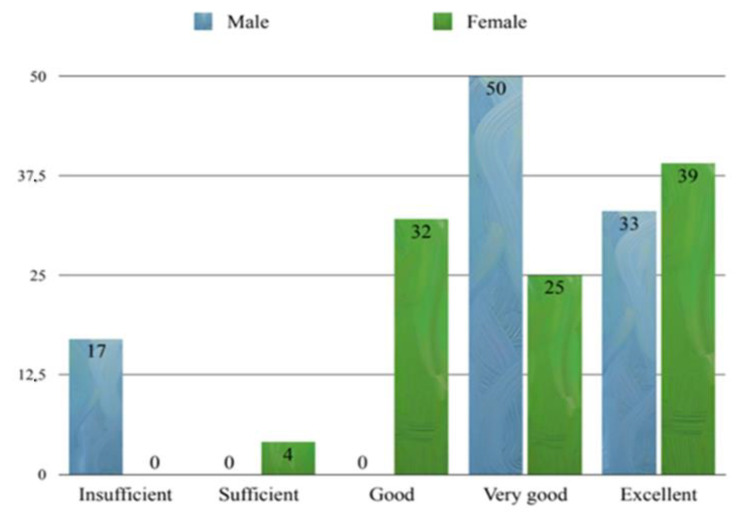
Distribution of grades (oral exam) according to gender.

**Figure 8:**
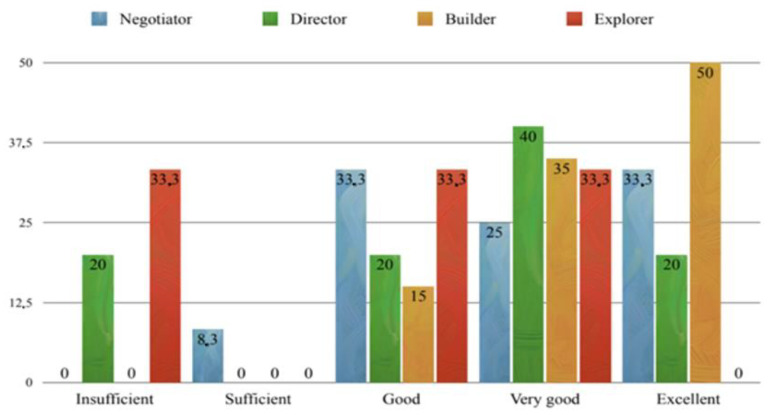
Distribution of grades (oral exam) by Fisher’s primary types of temperament.

The distributions of test scores (first exam, “Dermatovenereology”) by sex and by temperament type (Fisher’s) are shown in Figure 7 and Figure 8. A correlation between exam scores and stress levels (per PSS) was found, i.e., higher exam scores (better results) were associated with higher stress (Spearman’s ρ = 0.578467, *p* < 0.05). When looking at the impact of PMR on the students’ stress levels, a positive influence was seen. Thus, a positive correlation was observed between pre-exam cortisol levels and the more frequent application of PMR guidelines (Spearman’s ρ = 0.416138, *p* < 0.05), with a positive evaluation of these guidelines by students themselves (Spearman’s ρ = 0.363745, *p* < 0.05), and the cortisol level measured after non-exam period (Spearman’s ρ = 0.59228, *p* < 0.05).

## 4. Discussion

Earlier studies have analyzed the effect of acute stress during students’ oral exams (pre-exam stress, specifically) to study the stress paradigm, and they observed increased cortisol levels in anticipation of stressful situations [8,40,41]. In addition to oral exams [7,42,43,44,45,46,47,48,49], a significant source of stress for dental students are clinical exercises, competition with colleagues for grades, and the fear of failing an exam or the academic year [42,50,51]. Our research additionally supports/indicates the influence of certain other stress-related factors, such as gender, as significantly higher stress (PSS) was observed in women than men, which corresponds to many studies that have shown that the study of dental medicine is more stressful for women/students than for their male colleagues [43,50,51]. Although in earlier psychoneuroendocrine (PNE) studies women showed a lower response to stress [52,53], according to recent studies, women subjectively perceived experiencing greater stress and had a less favorable cognitive assessment (trouble remembering and learning new things, difficulty with everyday life decisions), likely related to their higher risk of developing mood disorders and anxiety [54,55,56]. Accordingly, recent research shows that women usually report more stress than men [57,58,59]. Although our study indicates significantly higher stress (PSS) in women than men, cortisol values did not significantly differ between the sexes. A few studies, though, observed higher cortisol levels in men than women, where cortisol was measured just before taking a written and oral exam [52,60,61]. A potential explanation is the fact that our respondents were only exposed to an oral and not a written exam. Also, when comparing groups by levels of perceived stress (PSS), low, medium, and high, there was no difference in participants’ pre-exam salivary cortisol levels, though it should be noted that high perceived stress was noted in the majority of our subjects/students (97.5%).

The comparison of our students’ pre-exam cortisol levels with their cortisol values during the non-exam period showed significantly higher pre-exam cortisol values. Our students’ strong cortisol increase in anticipation of the exam corresponds to several previous studies [11,41,62,63], which showed higher cortisol values before exams than during the period immediately after summer vacation. Most previous research results correspond to our results, showing decreased cortisol concentrations in a non-exam period compared to a pre-exam period [41,63,64]. Several studies, however, did not reveal changes in cortisol concentrations when comparing the two periods [65,66], and some studies even indicated an additional increase in cortisol concentration after the exam [23,67]. Although some studies have shown that cognitive assessments affect the cortisol response to stressors [68,69], our results did not prove a correlation between pre-exam stress and PSS, which is in line with previous research, which shows a weak and divergent connection between perceived stress and measured stress [70,71,72]. Another potentially important factor for a person’s experience and perception of stress can also be personality traits. By comparing students’ pre-exam cortisol levels by temperament type (according to Fisher’s division), no significant association was found, but some observations were noted: *Explorer* and *director* temperaments have lower average pre-exam cortisol values than *builder* and *negotiator* temperaments. According to previous research results, the *explorer* temperament (which was the least represented among the surveyed students) is linked to the neurotransmitters dopamine and noradrenaline, with certain behavioral patterns [32]. *Explorers* are characterized by creativity, curiosity, impulsiveness, and tendency toward risky activities and seeking excitement and novelty [32]. Thus, Fisher’s temperaments, like the curious and impulsive *explorer* and the extroverted and determined *director*, offer a clear picture of why these individuals’ cortisol levels would not increase very much in the pre-exam period. However, despite the promising results of some studies showing an association between personality factors and cortisol response, other studies like ours have failed to establish a consistent link [17,18,19].

In addition, we found a significant difference in stress levels between people of different characteristics. A significantly higher level of stress (the PSS) was seen in *builders*. According to Fisher and colleagues, the *director*’s character is linked to testosterone (based on earlier research findings on the role of testosterone in cognitive processes and socio-emotional engagement), where individuals with this pronounced *director* temperament are characterized by dominance and attention to detail [73]. Based on these findings, compared to women, men should be more analytical/decisive, which has been confirmed by previous studies [32,74]. On the other hand, the presumed biological basis of the *builder* temperament is the neurotransmitter serotonin [73]. So, the traits attributed to people with a pronounced *builder* dimension (based on previous research) include a connection to serotonin, pronounced numerical and pictorial creativity, pronounced sociability and self-control, as well as self-transcendence [32]. Also, according to earlier studies, lower neuroendocrine stress is associated with high self-esteem, high extroversion, low anxiety, and low neuroticism [75,76]. The above can explain the lower PSS level in our extroverted and decisive subjects (students) with the *director* temperament. Thus, we partially accept the assumption that PSS levels differ between the primary types of temperaments defined by Fisher’s inventory. Our results show an interesting connection (positive correlation) between students’ stress levels (PSS) and exam grades—the greater the student’s perceived stress, the higher the exam grade. Notably, students consider the “Dermatovenereology” exam used for this study one of the more demanding exams. This connection between grades and PSS level may indicate that these students take very seriously their studies and responsibilities for the courses they attend. Concerning temperament traits in relation to exam grades, *builders* received the most fives (excellent), while *directors* had the most fours (very good). This shows that the *builder* traits (numerical and pictorial creativity, pronounced sociability, and self-control) in comparison to *director* traits (attention to detail and lower level of verbal fluency) result in better *builder* success in oral exams. By gender, female students more often had the best grade (excellent, five), while men more often had a grade of very good (four). Numerous articles on stress during university studies have established that studying dental medicine is more stressful for female students than for their male colleagues [43,50,51]; our results also confirmed this, showing higher stress (PSS) in female students than male students. Our results related to PMR show that this technique was effective at reducing pre-exam stress. The participants who applied the PMR technique, and who had their cortisol measured a third time before an exam similar to the first, showed a drop in cortisol levels. This group included only 25% of participants who had the highest pre-exam (first exam) cortisol levels, and their drop in cortisol at subsequent measurements indicates that PMR provided a beneficial effect for these students. The three measurement time points were (1) just before the first exam; (2) during the non-exam period after summer break; and (3) just before the second exam, after applying PMR, and a significant drop in participants’ cortisol levels was observed over those time points. In fact, the lowest cortisol level was observed at the 3rd measurement point, indicating that applying the relaxation technique caused a significant reduction in pre-exam stress. These results confirm what other studies have found concerning stress in students before and after PMR [77,78,79,80]. Pv and Lobo [77] applied the PMR technique to 30 first-year nursing students (random sample) and found a significant reduction in stress. Also, Arabaci et al. [78] and Dehkordi et al. [79] proved that introducing PMR for students during their first clinical experiences helps to relieve stress and contributes to their professional development. Some studies have even recommended that PMR should accompany all formal nursing education in order to encourage greater satisfaction and a positive outlook on their study [80]. So, since a correlation between the pre-examination cortisol level and the frequency/using the PMR guidelines has been proven (k = 0.416138), it suggests that students with high pre-exam cortisol and high stress had a greater willingness to apply the guidelines. This shows that the students are aware of the stressful situation they are in and are willing to use relaxation methods to ease their stress, and at the same time work on themselves and their health. Therefore, we support the implementation of such useful methods in the curricula and practices of all biomedical studies, including medicine and dentistry, as these undergraduates often experience significant symptoms of stress due to the lengthy duration and demands of their studies [5,6,7].

Understanding the impact of AS on the organism and the complex relationship of stress-induced interactions between psychological and neuroimmunological factors can help identify students who are prone to AS. If they can be identified early on in their studies, they can be introduced to healthy coping methods before the possible onset of PNE changes that contribute to the development of stress-associated conditions (e.g., cardiovascular, autoimmune, and other diseases) [81,82,83,84]. According to the literature data so far, this population is susceptible to stress even after graduation, and therefore it is important to educate them on how to reduce the impact of stress on the body [21]. Since stress affects immune changes and is an important risk factor for many pathological conditions, reducing stress would not only benefit dental and medical students and professionals but also the entire communities they serve by creating a healthier, more stable healthcare system. Ours is the first study that looked at AS from the aspect of psychoneuroendocrinology, so it is necessary to continue research in this direction and find ways to make life easier for students during their university years and beyond. In recognizing the constraints of our investigation, we note primary aspects that might influence the interpretations and relevance of our outcomes concerning cortisol fluctuations and their association with anxiety among college attendees. Our study did not consider the differences in sleep habits among college students, despite the known significant impact of sleep on cortisol levels. Research consistently shows that cortisol responses are closely tied to both the amount and quality of sleep, with poor or fragmented sleep elevating cortisol levels, which in turn affects mental well-being and stress endurance. College students, especially during exam periods or project deadlines, might exhibit irregular sleep schedules due to prolonged study sessions or heightened stress, which could skew the observed relationship between stress, cortisol, and anxiety in our research. Omitting sleep as a variable fails to acknowledge a potent factor that could markedly alter cortisol behavior and its linkage with anxiety, thereby narrowing the applicability of our results to wider contexts or scenarios with more stable sleep patterns. Moreover, the direct correlation between ECTS points and the difficulty level of university subjects is complex and subject to various factors, including student background and teaching methodologies. Although the use of ECTS points offers a standardized framework for educational comparisons within and across European institutions, specific research directly linking ECTS points to the difficulty of university courses, or examining the perceived workload among students in different disciplines, has not been identified in recent literature. This gap highlights an area for further investigation. The absence of such research underscores the need for additional studies to explore the relationship between ECTS points and course difficulty, or student workload, more comprehensively. Despite the aforementioned and some other certain limitations (a relatively small number of participants, unicentric nature, and objective economic and financial circumstances), this research serves as a foundation for future multicentric studies with a larger number of participants.

## 5. Conclusions

The PSS-based group analysis revealed nearly all participants experiencing high stress, with women showing significantly higher stress levels than men. Notably, stress level differences were noted between *director* and *builder* temperaments. Following the implementation of PMR, there was a significant reduction in cortisol levels. The exclusion of sleep patterns as a variable introduces potential biases and overlooks crucial factors affecting cortisol levels and stress perception. This limitation, along with the study’s modest participant size and unicentric nature, suggests caution in generalizing the results too broadly. Nonetheless, our findings contribute to the understanding of stress management in academic settings, emphasizing the need for future research to include a broader array of variables and a larger, more diverse participant pool. This approach could enhance our comprehension of stress’s physiological and psychological impacts, guiding more effective stress management strategies.

## Data Availability

Data are available upon request to the corresponding author.
